# Association between blood lead levels and parathyroid hormone among United States adolescents aged 12–19: a cross-sectional study

**DOI:** 10.3389/fendo.2024.1383058

**Published:** 2024-07-09

**Authors:** Baomei He, Xiaowei Wang, Huanjun Luo, Qin Zhou

**Affiliations:** ^1^ Center for Reproductive Medicine, Department of Pediatrics, Zhejiang Provincial People’s Hospital (Affiliated People’s Hospital, Hangzhou Medical College), Hangzhou, China; ^2^ Bengbu Medical College, Bengbu, Anhui, China

**Keywords:** blood lead levels, parathyroid hormone, adolescents, national health and nutrition examination survey, vitamin D

## Abstract

**Aims:**

Studies on the association between serum lead levels and parathyroid function in adolescents are lacking. Therefore, in this study, we elucidated the possible association between blood lead levels (BLLs) and the parathyroid hormone (PTH) in adolescents aged 12–19 years in the United States.

**Methods:**

In this study, information from the database of the National Health and Nutrition Examination Survey was utilized. The study included 3919 participants from survey cycles between 2003–2004 and 2005–2006. Multivariable linear regression analysis was performed to determine the correlation between BLLs and PTH. Furthermore, smooth curve fitting was utilized to analyze the dose–response relationship between BLLs and PTH.

**Results:**

Multivariable linear regression analysis revealed that every 1 μg/dL increase in BLLs was associated with 0.67 pg/mL increase in PTH (β = 0.67, 95% CI: 0.16–1.18, p < 0.01). However, sex-stratified subgroup analysis revealed that this positive association was only observed in males (β = 1.16, 95% CI: 0.50–1.83 p < 0.01). Smooth curve fitting revealed a positive correlation between BLLs and PTH.

**Conclusions:**

In adolescents in the United States, BLLs are positively correlated with PTH, particularly in males.

## Introduction

Lead is a harmful heavy metal that is ubiquitous in the environment; its toxicity remains an important public health concern because populations are still exposed to low lead levels (1–3). As per the Centers for Disease Control and Prevention, there are no safe blood lead levels (BLLs) for children because lead exhibits toxicity even at low levels. Exposure to low levels of lead can exert deleterious effects on multiple organ systems in the human body (4, 5). Furthermore, prolonged lead exposure can result in detrimental health outcomes in adults, encompassing cardiovascular diseases, renal impairment, reproductive disorders, and neurological dysfunction (6–9). Moreover, in expectant women, lead exposure may lead to complications such as preterm birth or low birth weight (10, 11).

Exposure to lead in the environment during childhood and the resultant health issues are considered public health disasters (12). Compared with adults, children are at a higher risk of lead exposure because they possess enhanced absorption capabilities and limited excretion capacities (13). Lead exposure among children is associated with blood pressure, endocrine-disrupting activity, cognitive impairment, developmental delay, decreased intelligence quotient, and behavioral issues (14–16).

The parathyroid hormone (PTH) is a polypeptide that plays a key role in regulating calcium and phosphate levels in the body. In the parathyroid gland, PTH is synthesized and subsequently cleaved to exclusively generate the active state.

Studies have revealed that lead affects blood PTH levels. Kristal-Boneh et al. (16) and Potula et al. (17) confirmed the correlation between lead exposure in the workplace and levels of PTH in the bloodstream, noting a significant rise in PTH among individuals with occupational lead exposure. However, most studies are limited owing to their small sample size; furthermore, the findings are contradictory (18, 19). In addition, there is little information regarding the effects of serum lead on PTH levels in the general population with low-level exposure.

Research has indicated that lead disrupts the metabolism of vitamin D through its impact on the expression of metabolizing enzymes (20), and there is a negative correlation between BLL and free 25(OH)D, and a positive correlation between BLL and Vitamin D binding protein in healthy adolescents (21). These results suggest that lead may affect PTH levels through its involvement in vitamin D metabolism.

Increasing evidence indicates elevated lead levels in the general population. Therefore, further elucidating the possible association between BLLs and calcium homeostasis markers such as PTH is essential. Previous individual primary studies have presented inconclusive evidence of this relationship.

Considering limited studies in children and adolescents, in this current investigation, we investigated the potential correlation among exposure to low BLLs and PTH levels in children and teenagers residing in the United States. For this, we used data from the National Health and Nutrition Examination Survey (NHANES) from 2003 to 2006.

## Methods

### Study design

NHANES is a nationwide survey that collects information on the nutritional and health statuses of United States individuals who are not residing in institutions. This program started as a sequence of surveys during the early 1960s and has been continuously running since 1999. Around 5,000 people from different regions nationwide are included in this survey each year. This study was approved by the Ethics Review Board of the National Center of Health Statistics (NCHS). All participants provided their consent after being fully informed. Parents/guardians provided written informed consent for children under 18 years of age, while individuals aged 18 or above autonomously provided their own signature on the document.

### Study population

NHANES data from 2003–2004 to 2005–2006 were used because this was the only period when information on PTH measurements was collected. We only focused on individuals aged 12–19 years with information on blood PTH and lead levels. Among the eligible participants who met this criterion, 4,015 participants were included. However, 96 pregnant women were excluded from the final sample size; as a result, a cohort comprising 3,919 participants was selected for further examination.

### Measurement of BLLs and PTH

Blood samples were obtained during the physical examination, preserved at a freezing temperature of -20°C, and transported to a central laboratory. Immunological assays were performed to measure serum PTH levels. The protocols outlined on the NHANES website were used (Data Documentation, Codebook, and Frequencies, Parathyroid Hormone). Inductively coupled plasma mass spectrometry was used to measure lead levels in whole blood samples. We treated BLL concentrations as continuous variables and categorized them into quartiles: <0.63μg/dL, 0.63–0.90μg/dL, 0.9–1.36μg/dL, and ≥1.36μg/dL, respectively.

### Variables

The covariates analyzed were age, body mass index (BMI), serum calcium levels, cotinine levels, vitamin D levels, and estimated glomerular filtration rate (eGFR). Furthermore, sex, race/ethnicity, physical activities, and other relevant covariate acquisition processes available in the NHANES dataset (http://cdc.gov/nchs/nhanes) were included as categorical variables.

Race/ethnicity was self-reported by the participants. The participants were divided as follows: Mexican American, Other Hispanic, non-Hispanic white, non-Hispanic black, or others. BMI was calculated by dividing the weight in kilograms by the height in meters squared. The Chronic Kidney Disease Epidemiology Collaboration equation based on serum creatinine levels was used to determine eGFR (22). The accelerometer recorded the intensity and frequency of movement. Per-minute activity counts were calculated. The average weekly duration of moderate-to-vigorous physical activity (MVPA) was measured. MVPA was defined as counts per minute ≥ 2020 (23).

### Statistical analysis

R (http://www.R-project.org) and EmpowerStats (http://www.empowerstats.com) were used to perform statistical analyses. The significance level was p < 0.05. Sample weights were used according to the analytical guidelines provided by NCHS for estimating all values to ensure data representativeness for the civilian noninstitutionalized US population. Continuous variables are represented as mean (standard error) using a weighted linear regression model. Categorical variables are represented as % using the weighted chi-squared test. Three multivariable linear regression models were constructed: model 1, without any adjusted covariates; model 2, adjusted for age, sex, and race; and model 3, adjusted for all covariates presented in **Table 1**. In addition, subgroup analyses were performed. A weighted generalized additive model and smooth curve fitting were employed to account for potential linear relationships.

**Table 1:** Baseline characteristics of the study participants by quartiles of BLLs.

Characteristics	Quartile 1(N=966) (<0.63μg/dL)	Quartile 2(N=871) (≥0.63, <0.90μg/dL)	Quartile 3(N=1099) (≥0.90, <1.36μg/dL)	Quartile 4(N=983) (≥1.36μg/dL)	P value
Age, years	15.52 (0.11)	15.54 (0.12)	15.39 (0.16)	15.25 (0.13)	0.032
BMI, kg/m^2^	23.92 (0.31)	23.62 (0.30)	23.50 (0.30)	22.98 (0.25)	0.006
Cotinine, ng/Ml	8.08 (1.36)	21.71 (2.91)	27.46 (4.00)	37.48 (4.76)	<0.001
Calcium, mg/dL	9.70 (0.02)	9.73 (0.02)	9.77 (0.02)	9.78 (0.01)	<0.001
Vitamin D, nmol/L	64.50 (1.31)	63.41 (1.63)	62.22 (1.65)	60.97 (1.97)	0.006
eGFR, ml/min per 1.73 m^2^	127.20 (0.98)	127.38 (0.95)	130.00 (1.17)	134.29 (1.23)	<0.001
GENDER, %					<0.001
Male	33.84 (2.52)	45.28 (2.63)	62.21 (1.66)	74.37 (2.46)	
Famale	66.16 (2.52)	54.72 (2.63)	37.79 (1.66)	25.63 (2.46)	
RACE, %					<0.001
Mexican American	9.35 (1.54)	10.56 (1.41)	11.36 (1.82)	16.30 (2.68)	
Other Hispanic	3.09 (1.13)	3.43 (0.74)	5.74 (1.37)	8.09 (1.44)	
Non-Hispanic white	73.10 (2.47)	68.10 (3.49)	59.24 (3.57)	47.66 (4.58)	
Non-Hispanic black	9.88 (1.98)	13.34 (2.07)	17.10 (2.02)	22.70 (3.36)	
Other race/ethnicity	4.58 (1.08)	4.57 (1.35)	6.56 (1.28)	5.25 (1.13)	
Physical activity, %					<0.001
Sedentary	21.99 (3.12)	16.92 (2.53)	20.76 (2.74)	15.37 (2.60)	
Low	44.45 (2.68)	47.26 (2.86)	42.43 (2.97)	39.38 (3.88)	
Moderate	28.92 (3.23)	31.11 (3.03)	28.40 (3.00)	26.25 (3.59)	
High	4.65 (1.08)	4.71 (1.40)	8.40 (2.05)	19.00 (3.05)	

Continuous variables are represented as mean (standard error). p-values were calculated using a weighted linear regression model. Categorical variables are represented as %; p-values were calculated using the weighted chi-squared test.

## Results

### Participant selection

**Figure 1:**
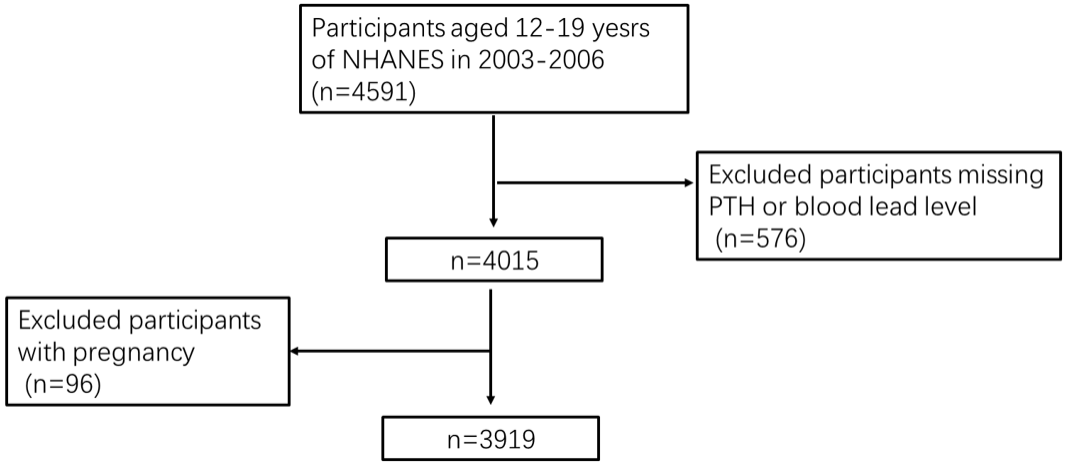
Flow chart of participants.

**Figure 1** illustrates the flow chart of participant selection. After excluding participants with missing information on blood PTH or lead levels, 576 adolescents aged 12–19 years were excluded. Furthermore, 96 pregnant women were excluded. Finally, 3,919 eligible adolescents were included in this study.

### Baseline characteristics

**Table 1** summarizes the demographic profiles of the participants in different BLL quartiles. Individuals with the highest BLLs were younger and predominantly male; they exhibited lower BMI and vitamin D levels. In contrast, they exhibited higher eGFR, cotinine levels, and calcium levels than those with the lowest BLLs.

### Association between BLLs and PTH

**Table 2** summarizes the correlation between blood PTH and BLLs. According to the non-adjusted model, BLLs and PTH levels show a positive association. Furthermore, the multivariable-adjusted model revealed a positive correlation between BLLs and PTH. Each 1 μg/dL increase in BLLs corresponded to a 0.67 pg/mL rise in PTH (β = 0.67, 95% CI: 0.16–1.18, p < 0.01). In addition, in both models 1 and 3, when considering blood lead as a categorical variable (quartiles), the participants in the highest BLL quartile exhibited higher PTH levels compared with those in the lowest quartile (Q3: β = 3.15, 95% CI: 1.07–5.24, p=0.003; Q4: β = 3.75, 95% CI: 1.61–5.90, p < 0.01 and Q3: β = 2.39, 95% CI: 0.32–4.47, p = 0.024; Q4: β = 2.87, 95% CI: 0.66–5.08, p = 0.011, respectively). Next, sex-stratified subgroup analysis was performed. A significant association was observed between BLLs and PTH levels in males, as indicated by consistent findings in all multivariable linear regression models. However, this association was not statistically significant in females (**Table 2**). Furthermore, race-stratified subgroup analysis revealed that this positive association persisted among Mexican American individuals (β = 1.16, 95% CI: 0.11–2.20, p = 0.031), non-Hispanic white individuals (β = 1.09, 95% CI: 0.10–2.08, p = 0.032), and non-Hispanic black groups (β = 1.57, 95% CI: 0.15–3.00, p = 0.031).

**Table 2:** Association between BLLs and PTH levels.

	Model 1, β (95% CI)	Model 2, β (95% CI)	Model 3, β (95% CI)
BLL, µg/dL	0.57 (0.05, 1.10) 0.030	0.21 (-0.31, 0.73) 0.424	0.67 (0.16, 1.18) <0.010
Quintiles Q1 (<0.63)	Reference	Reference	Reference
Q2 (≥0.63, <0.90)	1.42 (-0.79, 3.63) 0.207	0.64 (-1.55, 2.84) 0.566	0.67 (-1.46, 2.80) 0.536
Q3 (≥0.90, <1.36)	**3.15 (1.07, 5.24) 0.003**	1.57 (-0.56, 3.69) 0.148	**2.39 (0.32, 4.47) 0.024**
Q4 (≥1.36)	**3.75 (1.61, 5.90) <0.001**	1.22 (-1.02, 3.46) 0.286	**2.87 (0.66, 5.08) 0.011**
P for trend	0.000	0.205	0.004
Stratified by gender			
Male	**1.08 (0.40, 1.76) 0.002**	**0.77 (0.10, 1.44) 0.024**	**1.16 (0.50, 1.83) <0.001**
Famale	1.27 (-0.29, 2.83) 0.110	-0.26 (-1.83, 1.31) 0.742	0.54 (-1.02, 2.09) 0.499
Stratified by race			
Mexican American	0.59 (-0.45, 1.64) 0.265	0.60 (-0.47, 1.66) 0.270	**1.16 (0.11, 2.20) 0.031**
Other Hispanic	-0.02 (-3.04, 2.99) 0.987	-0.29 (-3.47, 2.90) 0.859	0.17 (-3.12, 3.47) 0.919
Non-Hispanic white	0.84 (-0.16, 1.84) 0.099	0.85 (-0.15, 1.86) 0.097	**1.09 (0.10, 2.08) 0.032**
Non-Hispanic black	0.76 (-0.66, 2.19) 0.293	0.36 (-1.11, 1.82) 0.633	**1.57 (0.15, 3.00) 0.031**
Other race/ethnicity	-1.21 (-6.86, 4.45) 0.676	-2.07 (-7.89, 3.75) 0.486	-2.94 (-8.55, 2.66) 0.305

Model 1: No covariates were adjusted.

Model 2: Age, gender, race were adjusted.

Model 3: Age, gender, race/ethnicity, body mass index, physical activities, estimated glomerular filtration rate, serum calcium, vitamin D and cotinine adjusted. Bold fonts mean statistically significant.

**Figure 2:**
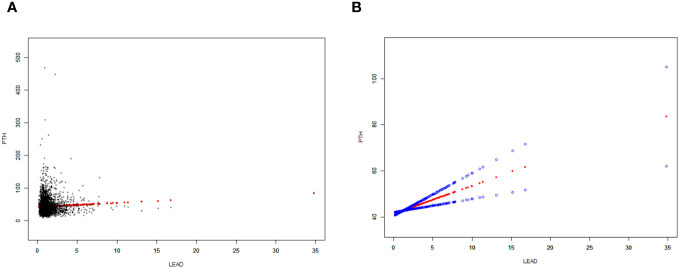
Correlation between BLLs and PTH levels. **(A)** Each data point is represented using a black dot. **(B)** The smoothed curve fit between the variables is represented by a solid red line, whereas the 95% confidence interval derived from the fit is indicated by the blue bands. Age, gender, race/ethnicity, body mass index (BMI), physical activities, estimated glomerular filtration rate (eGFR), serum calcium, vitamin D and cotinine were adjusted.

**Figure 3:**
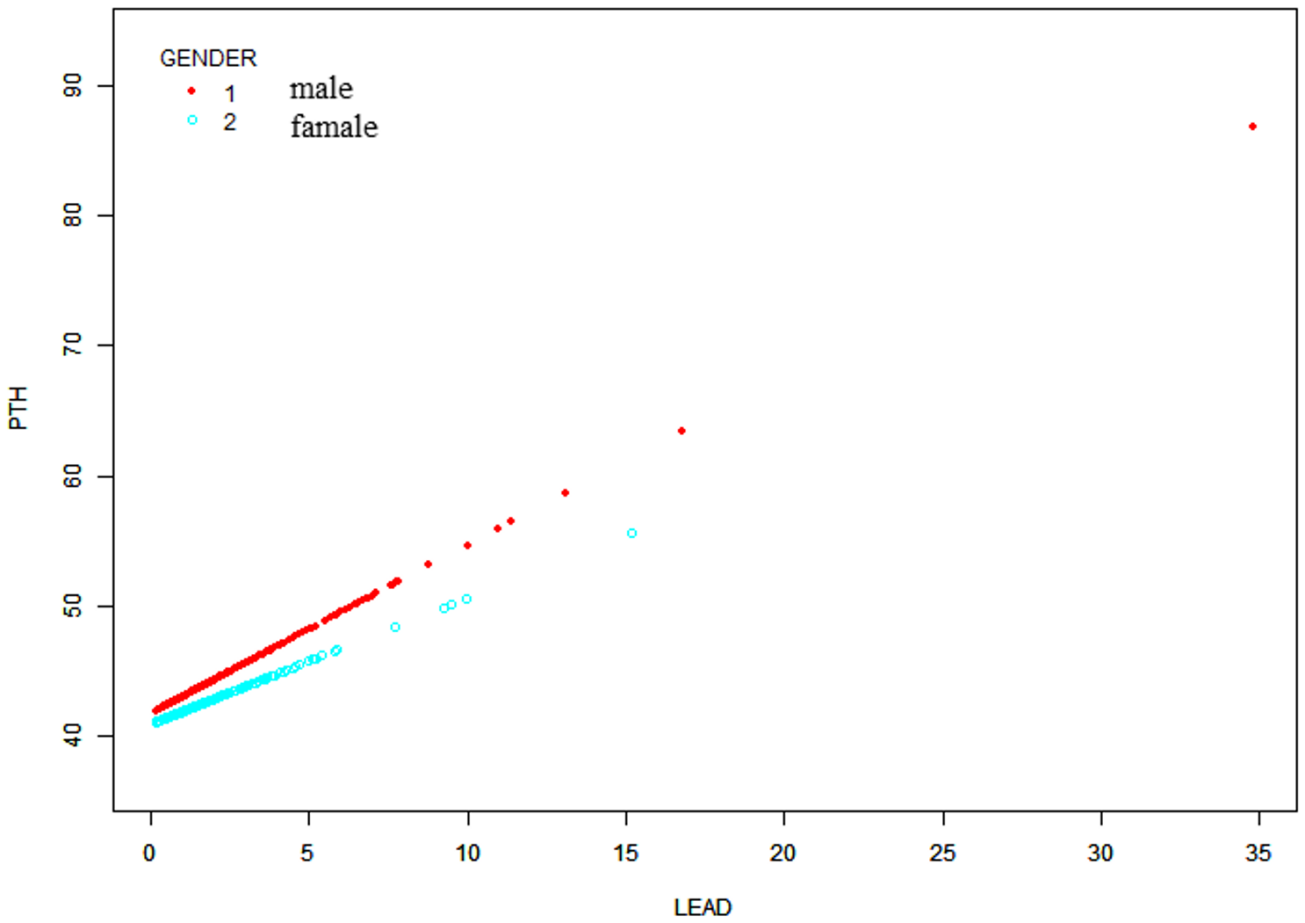
Sex-stratified association between BLLs and PTH levels. Age, race/ethnicity, BMI, physical activity, eGFR, serum calcium, vitamin D and cotinine were adjusted.

Moreover, a weighted generalized additive model was employed to consider the linear association and verify the results, while employing techniques for fitting smooth curves. (**Figures 2**, **3**). We further conducted analysis by excluding 8 subjects with serum lead levels ≥10μg/dL, and found that the results remained the same (**Supplementary Figures 1**, **2**).

## Discussion

In the present study, we utilized data from a representative sample of US adolescents aged 12–19 years to elucidate the potential association between BLLs and serum PTH levels. We observed that BLL is positively associated with PTH. Their relationships remained significant after adjustment for confounding factors. However, the potential role may differ by gender. We observed linear relationships between them, our multivariable linear regression analysis showed similar trends. Furthermore, race-stratified subgroup analysis revealed that this positive association persisted among most racial groups, including Mexican American, non-Hispanic white, and non-Hispanic black groups.

Research on the impact of lead on the endocrine system mainly focuses on individuals who are exposed to lead in their occupation and animal models used in experiments (24–27). In an 18-month (January 2017–July 2018), cross-sectional, case–control study, PTH levels and BLLs were significantly increase in 90 lead-exposed participants compared with controls (19). Similarly, in a cross-sectional survey including 146 people, Kristal-Boneh et al. reported that participants with occupational lead exposure exhibited substantial compensatory increases in PTH levels (16). Notably, the association demonstrated statistical significance among males while not achieving significance among females. In addition, a meta-analysis reported alterations in PTH levels following exposure to lead in the workplace (18). The pooled results revealed that the lead-exposed group had lower PTH levels than the control group. However, these results did not reach statistical significance, with unacceptable heterogeneity levels.

A NHANES study revealed that lead is positively associated with PTH in the general population aged ≥18 years (28). However, another study involving 105 children concluded that children with sufficient nutritional status and low-to-moderate lead exposure do not exhibit significant changes in vitamin D metabolism, as well as calcium and phosphorus balance, along with bone mineral content (29). In the present study, we revealed a stable positive association between BLLs and PTH in males aged 12–19 years.

The potential impact of lead exposure on parathyroid function may vary depending on gender. Baecklund et al. (30) revealed that BLLs were lower in women than in men (median 24 vs. 30 µg Pb/L). These sex disparities can be attributed to differences in calcium metabolism and calmodulin, renal sensitivity, iron storage status, and genetic factors between males and females (31, 32). Nevertheless, additional studies are warranted to examine the possible mechanisms or factors.

In the body, PTH is a central regulator of calcium homeostasis and plays a vital role in bone metabolism. It governs serum calcium levels by affecting the bones, kidneys, and intestine; however, its levels are regulated by the feedback mechanism of calcium levels (33). PTH stimulates calcium release from the bones into the bloodstream. Furthermore, it affects calcium reabsorption to enhance renal retention. Elevated BLLs may disrupt the hydroxylation of 25OH-vitamin D in the kidneys via the 1-α hydroxylase enzyme, resulting in the decreased production of active vitamin D [1,25 (OH)2D] (34). This subsequently results in decreased calcium levels.

Therefore, lead has the potential to act as a disruptor of the endocrine system and contribute to hormonal imbalances (15, 18, 24, 28). In this study, elevated BLLs could have decreased vitamin D levels, which, in turn, decreased serum calcium levels and increased PTH levels among lead-exposed individuals. However, our research revealed a positive correlation between lead levels and serum calcium levels, contradicting the previously mentioned mechanism by which lead affects PTH via serum calcium.

Our study has many strengths. First, a positive relationship was observed between BLL and PTH in adolescents; this has not been reported in prior research. Second, we examined a more extensive and representative multiracial population sample obtained from the NHANES, with rigorous measures and adjustment for important covariates.

Nevertheless, it is crucial to acknowledge the constraints of this study. Firstly, given its cross-sectional design, we cannot draw any causal inferences between blood lead levels (BLL) and parathyroid hormone (PTH). Secondly, the NHANES dataset does not include information regarding lead concentrations in bones, a commonly utilized measure for assessing cumulative lead exposure. In children, lead is primarily accumulated in trabecular bone, and its turnover rate is high (35).

## Conclusions

BLLs are positively correlated with serum PTH levels in adolescents aged 12–19 years, particularly in males.

## Data availability statement

Publicly available datasets were analyzed in this study. This data can be found here: http://cdc.gov/nchs/nhanes.

## Ethics statement

The studies involving humans were approved by the Ethics Review Board of the National Center of Health Statistics (NCHS). The studies were conducted in accordance with the local legislation and institutional requirements. Written informed consent for participation in this study was provided by the participants’ legal guardians/next of kin.

## Author contributions

BH: Writing – original draft. XW: Validation, Writing – review & editing. HL: Writing – review & editing. QZ: Writing – original draft.
