# Supplementary material for: Association between blood lead levels and parathyroid hormone among United States adolescents aged 12–19: a cross-sectional study
Source: Front Endocrinol (Lausanne). 2024 Jul 9;15:1383058. doi: 10.3389/fendo.2024.1383058 (PMC11263011; doi:10.3389/fendo.2024.1383058)
Supplement: Supplementary file 1 [file Table_1.docx]

Supplementary Table 1 Association between BLLs and PTH levels.

| BLL, µg/dL | β (95% CI)  male | famale |
| --- | --- | --- |
| Quintiles 1  Quintiles 2 | Reference  1.89 (-1.90, 5.68) 0.33 | Reference  0.15 (-2.27, 2.58) 0.90 |
| Quintiles 3 | 2.54 (-0.94, 6.01) 0.15 | 2.95 (0.42, 5.48) 0.03 |
| Quintiles 4 | 4.00 (0.48, 7.51) 0.03 | 1.68 (-1.26, 4.63) 0.26 |
| P for trend | 0.02 | 0.06 |

Age, gender, race/ethnicity, body mass index, physical activities, estimated glomerular filtration rate, serum calcium, vitamin D and cotinine adjusted.
