# Supplementary figures and images for: Association between blood lead levels and parathyroid hormone among United States adolescents aged 12–19: a cross-sectional study
Source: Front Endocrinol (Lausanne). 2024 Jul 9;15:1383058. doi: 10.3389/fendo.2024.1383058 (PMC11263011; doi:10.3389/fendo.2024.1383058)

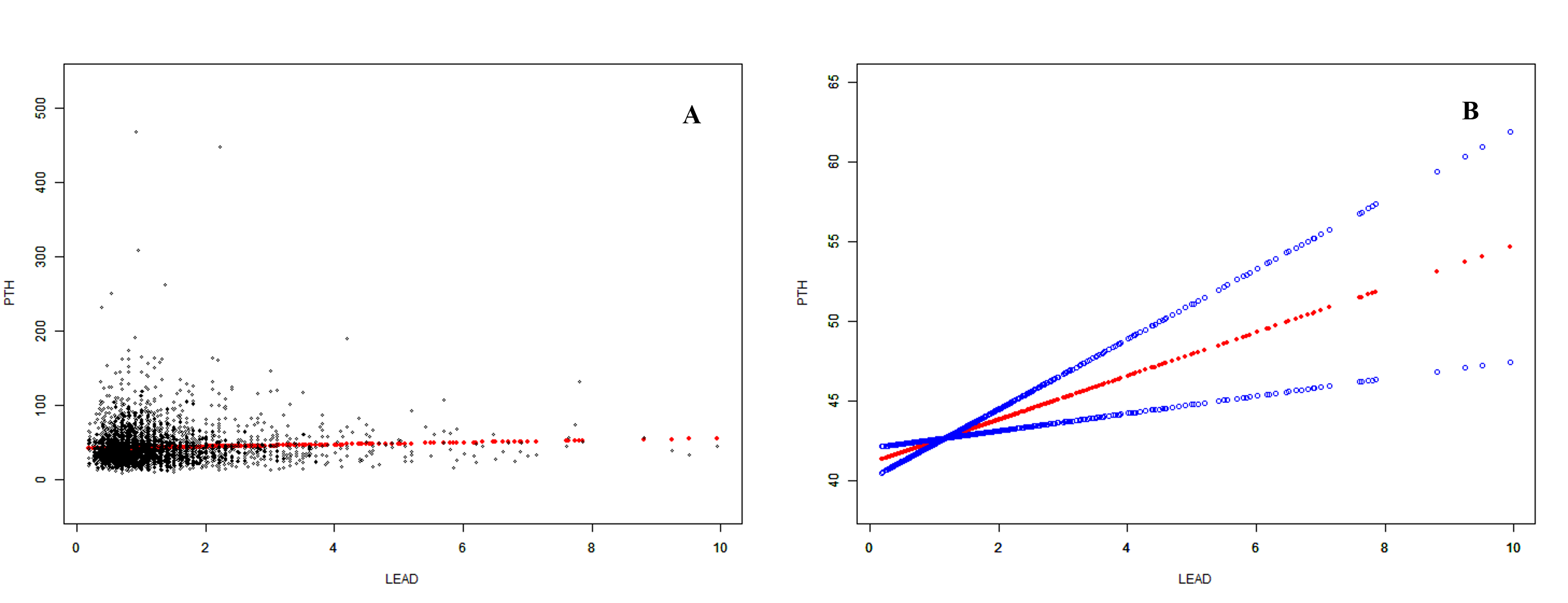

Supplement: Supplementary file 2 [file Image_1.tif]

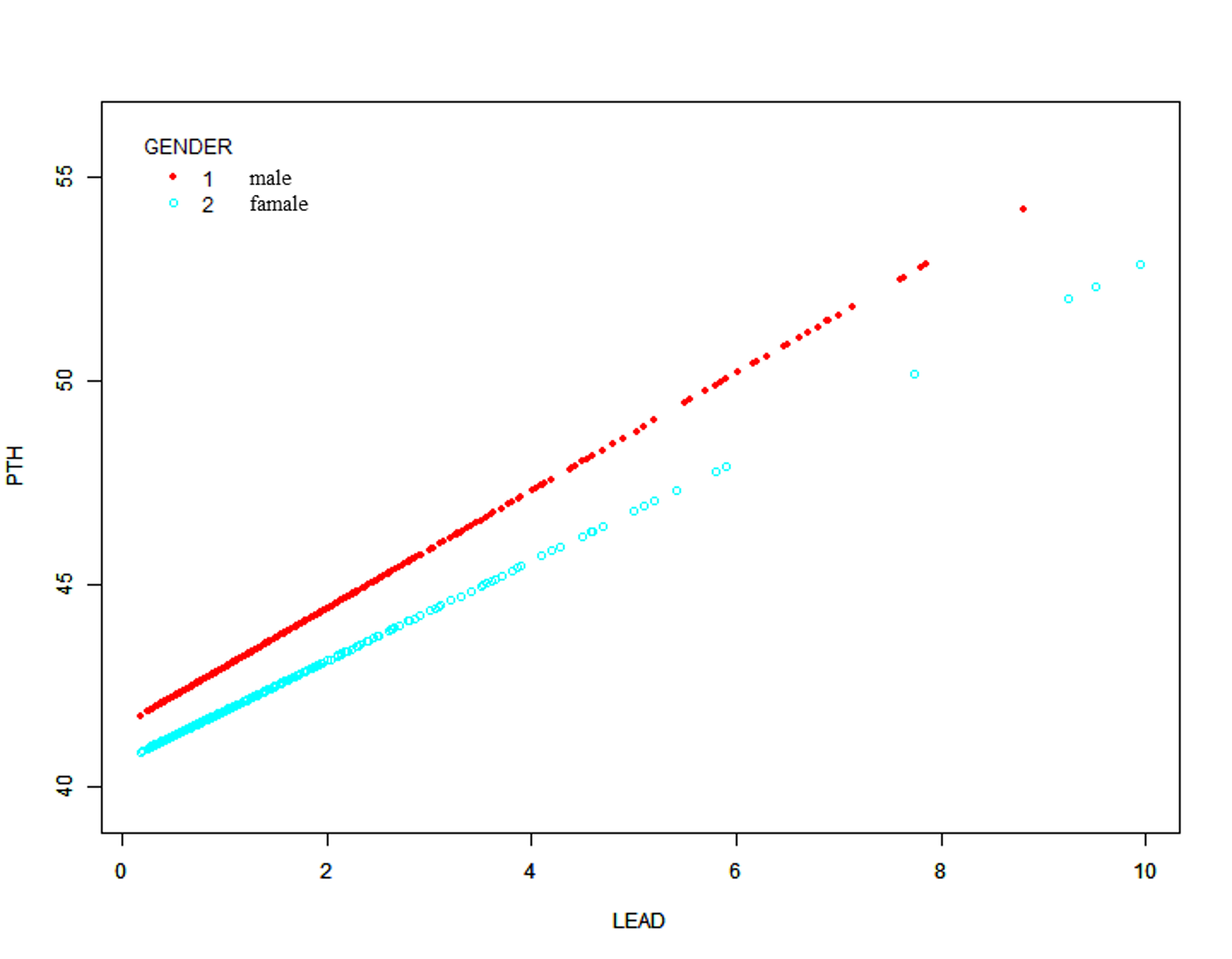

Supplement: Supplementary file 3 [file Image_2.tif]
